# Prenatal second-hand smoke exposure and the risk of suspected developmental coordination disorder in preschoolers: A nationwide retrospective cohort study in China

**DOI:** 10.3389/fpubh.2022.993471

**Published:** 2022-11-08

**Authors:** Meiqin Wu, Gareth J. Williams, Guixia Chen, Lan Zhang, Chunping Hu, Xiaotian Dai, Wenchong Du, Jing Hua

**Affiliations:** ^1^Shanghai Key Laboratory of Maternal Fetal Medicine, Department of Women's and Children's Health Care, Shanghai First Maternity and Infant Hospital, School of Medicine, Tongji University, Shanghai, China; ^2^School of Social Sciences, Nottingham Trent University, Nottingham, United Kingdom; ^3^Department of Children Healthcare, Women and Children's Hospital, School of Medicine, Xiamen University, Xiamen, China; ^4^Chengdu Women's and Children's Central Hospital, School of Medicine, University of Electronic Science and Technology of China, Chengdu, China; ^5^School of Public Health, Shanghai Jiao Tong University School of Medicine, Shanghai, China; ^6^Department of Psychology, Nottingham Trent University, Nottingham, United Kingdom

**Keywords:** second-hand smoke, developmental coordination disorder, retrospective cohort study, pregnancy, preschooler

## Abstract

Prenatal exposure to second-hand smoke (SHS) is associated with increased neurodevelopmental problems in children, however, its impact on the risk of developmental coordination disorder (DCD) in preschoolers have not been studied thoroughly. Herein, we probed this association based on a nationwide retrospective cohort study of 149,005 preschoolers in China. We divided the objects into the prenatal SHS-exposed group or the no prenatal smoke exposed group (NS-exposed group). Preschoolers were assessed for motor proficiency by the Chinese version of Little Developmental Coordination Disorder Questionnaire (LDCDQ). Multivariable logistic regression was used to evaluate the associations. The prevalence of prenatal SHS exposure was 23.89%. Generally, the prevalence of suspected DCD was significantly higher in prenatal SHS-exposed group (16.38% VS. 14.19%, *P* < 0.001). With the increase of age, the mean total scores of LDCDQ of both boys and girls increased gradually; and the prevalence of suspected DCD in girls was higher than that in boys in the same age group. After adjusting for covariates, prenatal SHS exposure had the negative association with the total score of LDCDQ and increased the risk of suspected DCD. Our results suggest a need for interventions designed to reduce maternal SHS exposure during pregnancy, early screen for DCD and increase targeted movement and coordination skill training for vulnerable children.

## Introduction

Tobacco smoke that remains in the environment by active smokers is called second-hand smoke (SHS) (1). SHS is a neglected public health issue that is responsible for the death of 880,000 people worldwide every year (2, 3). Women and children in developing countries and from lower socio-economic areas are at an increased risk of exposure to SHS due to factors including gender differences in the prevalence of smoking and poorly ventilated housing conditions (4).

A number of studies have documented a high prevalence of SHS exposure among pregnant women in developing countries, which can be as high as 93% in Vietnam (5) and 87% in India (6), and 45% in Mongolia (7). In China, the reported prevalence of SHS exposure varies, from 39 to 75% (8). However, these reports were derived from regional surveys and no national data in China is available yet. It is a known fact that smoking harms maternal and fetal health due to the diffusion of nicotine into fetal blood, amniotic fluid, and breast milk, which has negative effects on neurological development (9). Similar to smoking, SHS can also increase the risk of miscarriage, congenital malformations and stillbirths, lower mean birth weight, heart disease, lung cancer, and maternal depression (1).

Prenatal SHS exposure has also been associated with poorer physical outcomes for infants and children including low birth weight (10, 11), preterm birth (12, 13), and asthma (14, 15). Over recent years, a growing number of studies have demonstrated that the negative impact of prenatal SHS exposure extends beyond the physical health of infants to mental health and neurodevelopmental disorders, such as attention deficit hyperactivity disorder (ADHD) (16), behavioral problems (17, 18), language difficulties (19), and learning disabilities (20).

Maternal active smoking during pregnancy has been confirmed as a risk factor for developmental coordination disorder (DCD) (21). However, the relationship between prenatal SHS exposure and DCD in preschoolers has been rarely investigated. DCD is a neurodevelopmental disorder that occurs in 5–6% of children (22). Children with DCD are characterized by significantly impaired function in motor coordination; however, the etiology of the disorder is still largely unclear. An earlier study with a small sample (*N* = 122) (23), as well as another study involved 8,586 children in Shanghai, China, looking at maternal exposure to first- and second-hand smoking (24), both reported a lower risk of DCD in children whose mothers were not exposed to tobacco smoke during pregnancy. Nevertheless, there is no large-scale specific research on the relationship between prenatal SHS and DCD to confirm the impact of prenatal SHS exposure on off-spring neurodevelopment.

This current study aimed to use a large-scaled national representative population sample to investigate the association between prenatal SHS exposure and suspected DCD in preschoolers, adjusting for a wide range of potential confounders. So that appropriate health guidance can be provided for families and clinicians to mitigate risks associated with childhood motor impairment.

## Methods

### Study population

**Figure 1:**
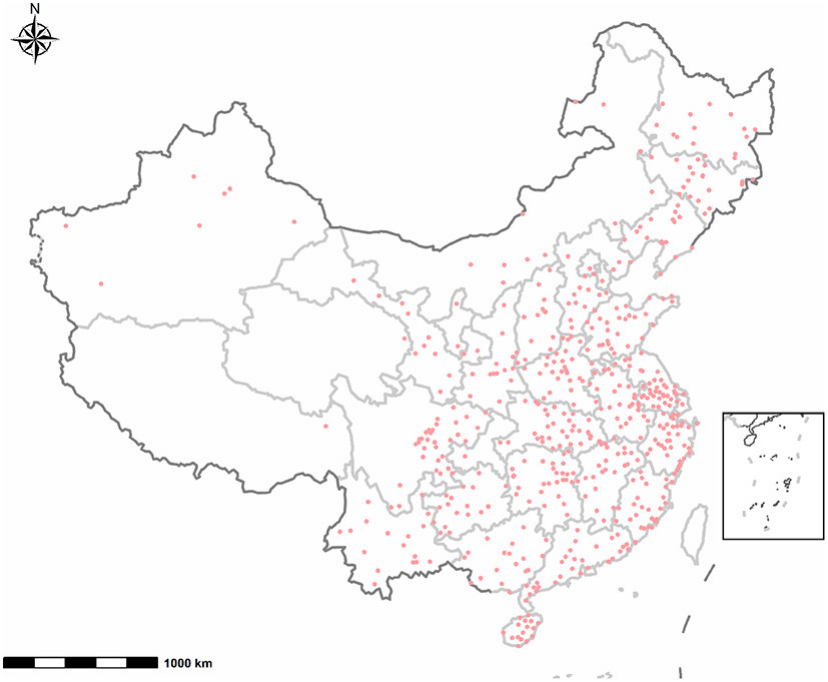
Distribution of 551 participant cities in mainland China.

**Figure 2:**
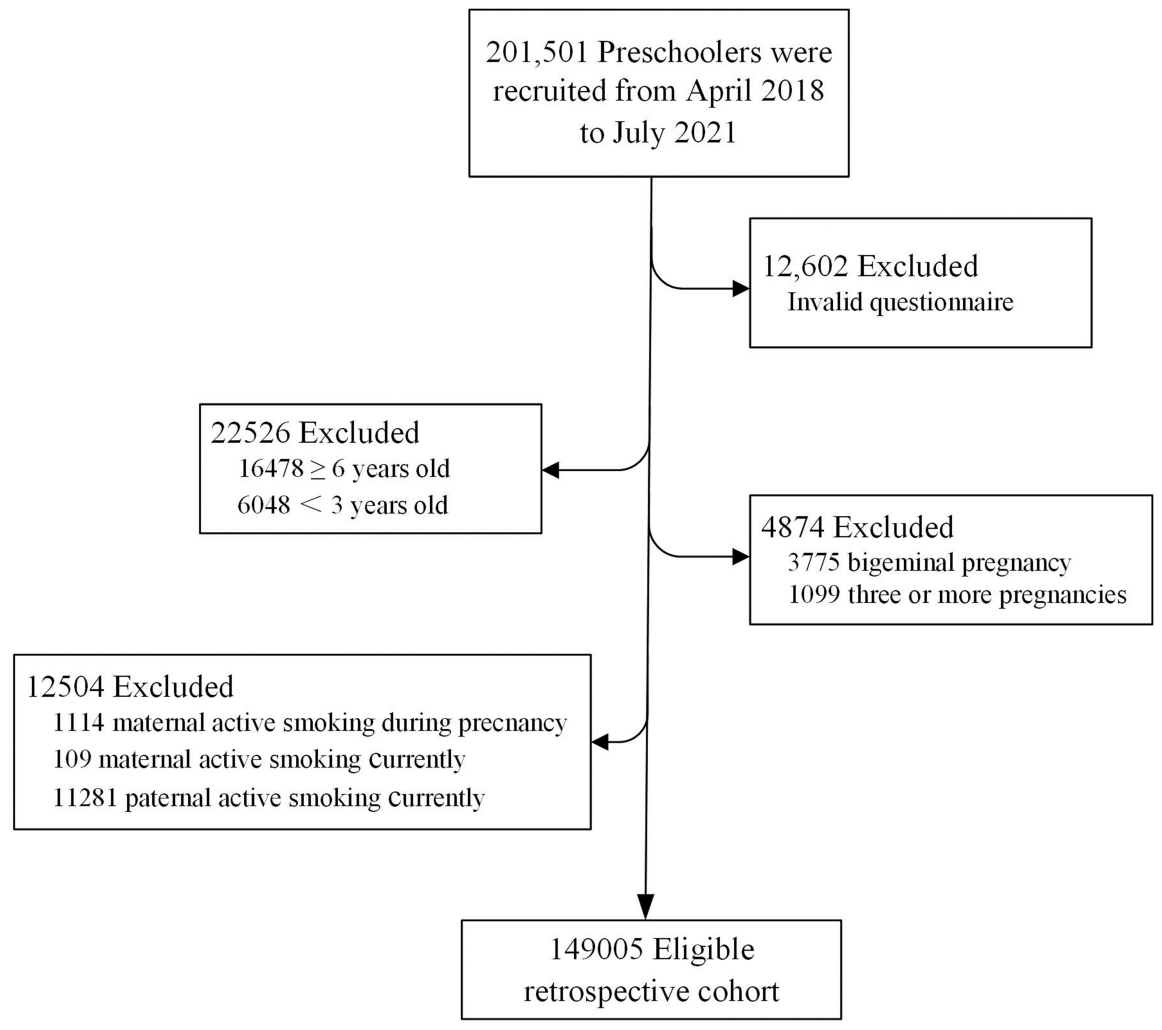
Flowchart of the study population.

A stratified cluster sampling plan was used to ensure that the participants included in the current study were representative of the Chinese population. China's 2018 to 2019 National Census data provided the basis for the stratification by geographic region, age, sex, and socioeconomic status. The government-supported maternity and children's health center in each city was selected to invite their local kindergartens to participate in the study. From April 2018 to July 2021, a total of 201,501 preschoolers were recruited from 2,503 public kindergartens in 551 cities from 31 provinces/municipalities/autonomous regions in mainland China (as shown in Figure 1). The children with no physical disabilities or intellectual impairment (according to the physical examinations prior to starting kindergarten) and their parents who had successfully submitted electronic questionnaires were included in the study. Then children with invalid questionnaires, children ≥ 6 years old or <3 years old, multiple births were excluded. We further excluded records with maternal active smoking during pregnancy, maternal or paternal active smoking currently. Finally, 149,005 singletons birth records were included for the final analysis. Figure 2 describes subject selection process. The current study was approved by the Ethics Committee of Shanghai First Maternity and Infant Hospital, School of Medicine, Tongji University (NO. KS18156). Each parent signed an informed consent before the investigation. More details on the survey and dataset have been described and published elsewhere (25, 26).

### Measures

#### Outcome

Children's motor performance was assessed using the Little Developmental Coordination Disorder Questionnaire (LDCDQ). The LDCDQ was developed to screen for motor coordination difficulties in children aged 3 and 4 years (27, 28), and it can also be extended for use with children as old as 5 years (29). The LDCDQ consists of 15 items divided into three subcategories: control during movement (CDM), fine motor skills (FM), and general coordination (GC). Each category contains five items; for each item, parents are asked to compare the performance of their child with that of children of the same age and sex, and to rate their child's performance on a 5-point Likert scale 1 point, not at all relevant to my child, to 5 points, extremely relevant to my child). Each subcategory has a maximum score of 25 points. Scores are summed to give a maximum total score of 75 points, with higher scores indicating a higher level of motor proficiency (29). The Chinese version of the LDCDQ has high internal consistency, good split-half reliability, and fair factor construct validity (30, 31), which following previous recommendations (27, 28). This study used the age- and sex-specific norms of the LDCDQ and cutoff scores were based on the previous study in China to indicate suspected motor impairments: suspected DCD was defined as LDCDQ scores ≤15th percentile; probably not DCD was defined as LDCDQ scores >15th percentile. Please refer to the published article (25) for details.

#### Exposure

We defined prenatal SHS exposure as exposure to secondhand tobacco smoke during the pregnancy in either one of two environmental settings: the mother's home; or the mother's workplace (if the main working space was indoors and if the mother stayed at work until the last month of pregnancy). Children were then divided into a group exposed to SHS and a group not exposed to smoke (NS).

#### Covariates

Information on covariates was also obtained through the self-reported questionnaire of participants (mother preferable). We included parental, neonatal and child characteristics as potential confounders according to previous research literature (25, 32) (Table 1). (1) Parental characteristics were maternal and paternal age at birth; parity (1, primiparous; 2, parous, second delivery; 3, parous, third and above delivery); maternal comorbidities and pregnancy complications (yes/no); level of maternal and paternal education and household incomes. (2) Neonatal characteristics were sex; gestational age; cesarean delivery (yes/no); birth weight; neonatal asphyxia; and neonatal intensive care unit (NICU) admission (yes/no). (3) Child characteristics were current age; body mass index (BMI, calculated as weight in kilograms divided by height in meters squared); medical history of movement disorders and psychiatric (yes/no); postnatal exposure to SHS (yes/no).

**Table 1:** Maternal, family and child characteristics of prenatal SHS-exposed and NS-exposed groups.

**Variable**		***N*** **(%)/*****M*** **(SD)**		***P***
		SHS-exposed group* **(*n* = 35,603)**	NS-exposed group* **(*n* = 113,402)**	
**Parental characteristics**				
Maternal age at birth, y				
	<20	1,036 (2.91)	2,219 (1.96)	<0.001
	20–29	26,320 (73.93)	78,182 (68.94)	
	30–39	7,999 (22.47)	31,938 (28.16)	
	≥40	248 (0.69)	1,063 (0.94)	
**Maternal education, y**				
	≤6	826 (2.32)	2,224 (1.96)	<0.001
	7–9	6,587 (18.50)	18,366 (16.20)	
	10–12	8,696 (24.42)	24.043 (21.20)	
	13–16	18,477 (51.90)	61,668 (54.38)	
	≥17	1,017 (2.86)	7,101 (6.26)	
**Parity, No**.				
	1	29,034 (81.55)	90,656 (79.94)	<0.001
	2	3,819 (10.73)	8,440 (7.44)	
	≥3	1,401 (3.94)	2,721 (2.4)	
**Maternal comorbidities and pregnancy complications**				
	Yes	33,532 (94.18)	107,662 (94.94)	<0.001
	No	2,071 (5.82)	5,740 (5.06)	
**Paternal age at birth, year**				
	<20	490 (1.38)	1,112 (0.98)	<0.001
	20–29	22,598 (63.47)	64,491 (56.87)	
	30–39	11,340 (31.85)	43,396 (38.27)	
	≥40	1,175 (3.30)	4,403 (3.88)	
**Paternal education, y**				<0.001
	≤6	735 (2.06)	1,585 (1.40)	
	7–9	7,182 (20.17)	17,072 (15.05)	
	10–12	10,012 (28.12)	24,629 (21.72)	
	13–16	16,579 (46.57)	61,047 (53.83)	
	≥17	1,095 (3.08)	9,069 (8.00)	
**Household incomes, CNY/Year/person**				
	<10,000	3,226 (9.06)	8,285 (7.31)	< 0.001
	10,000–29,999	4,120 (11.57)	10,708 (9.44)	
	30,000–49,999	4,075 (11.45)	11,117 (9.80)	
	50,000–99,999	4,319 (12.13)	14,145 (12.47)	
	100,000–149,999	2,415 (6.78)	9,394 (8.28)	
	150,0000–299,999	1,777 (4.99)	8,081 (7.13)	
	300,000–999,999	668 (1.88)	4,390 (3.87)	
	>1000,000	76 (0.21)	476 (0.42)	
	Don't know	6,383 (17.93)	17,526 (15.45)	
	Refuse	8,544 (24.00)	29,280 (25.82)	
**Neonatal characteristics**				
**Gestational age, wk**				
	<37	4,901 (13.77)	14,504 (12.79)	<0.001
	37–40	23,906 (67.15)	78,470 (69.2)	
	>40	6,796 (19.09)	20,428 (18.01)	
**Cesarean delivery**				
	Yes	16,994 (47.73)	52,785 (46.55)	<0.001
	No	18,609 (52.27)	60,617 (53.45)	
**Birth weight, g**				
	<2,500	1,589 (4.46)	5,622 (4.96)	<0.001
	2,500–4,000	31,812 (89.35)	100,747 (88.84)	
	>4,000	2,202 (6.18)	7,033 (6.20)	
**Neonatal asphyxia**				
	Yes	973 (2.73)	2,677 (2.36)	<0.001
	No	34,630 (97.27)	110,725 (97.64)	
NICU admission*				
	Yes	3,906 (10.97)	10,751 (9.48)	<0.001
	No	31,697 (89.03)	102,651 (90.52)	
**Child characteristics**				
Sex				
	Boy	18,567 (52.15)	59,452 (52.43)	0.367
	Girl	17,036 (47.85)	53,950 (47.57)	
	Age, y	4.47 ± 0.79	4.52 ± 0.78	<0.001
	BMI*, kg/m^2^	15.74 ± 2.15	15.80 ± 2.44	<0.001
**Medical history of movement disorders**				
	Yes	297 (0.83)	771 (0.68)	<0.001
	No	33,957 (95.38)	101,046 (89.10)	
	Missing	1,349 (3.79)	11,585 (10.22)	
**Psychiatric medication history**				
	Yes	347 (0.97)	736 (0.65)	< 0.001
	No	33,907 (95.24)	101,081 (89.14)	
	Missing	1,349 (3.79)	11,585 (10.22)	
**Postnatal exposure to SHS** ^**#**^				
	Yes	35,537 (31.34)	26,240 (73.70)	<0.001
	No	48,093 (42.41)	5,251 (14.75)	
	Missing	29,772 (26.25)	4,112 (11.5)	

* NS, No smoke; SHS, second hand smoke; NICU, neonatal intensive care unit; BMI, body mass index.

# Family members other than parents smoked.

### Statistical analysis

Group differences between SHS exposed and NS exposed groups, as categorical variables, were evaluated with the χ^2^-test, and group differences in continuous variables were examined by the independent samples *t*-test. The variance inflation factor (VIF) for each explanatory variable was used to identify the correlation between independent variables and the strength of the correlation (Supplementary Table 1). Multiple linear regression models were used to examine the main effects of prenatal SHS exposure on preschoolers' sub and total scores of the LDCDQ. Multivariable logistic regression was used to estimate the odds ratio (OR) and 95% CIs for the associations between SHS exposure and motor measurements and the risk of suspected DCD. We then fitted 3 levels of confounding adjustment models, and derived the adjusted odds ratio (AOR): model 2 adjusted for parental and perinatal characteristics; model 3 additionally adjusted for neonatal characteristics; and model 4 further adjusted for children's individual characteristics. An interaction term between prenatal SHS exposure and postnatal SHS exposure was constructed (Supplementary Table 2) and added to model 4. Subgroup analyses were performed to further control for the effects of postnatal SHS exposure.

To assess the robustness of the results, sensitivity analyses were conducted. According to previous studies gender, BMI score, preterm birth, and some prenatal conditions are significant risk factors for suspected DCD. We applied restrictions to yield a more homogeneous population with the following characteristics: maternal age at delivery was 20 to 35 years; maternal and paternal education period was 7–16 years; parity <3; no recorded maternal comorbidities and pregnancy complications; gestational age was between 37 and 40 weeks; birth weight was 2,500-4,000g; no recorded neonatal asphyxia and residence history of NICU; current BMI of <18; no medical history of movement disorders or psychiatric medication during preschool.

The statistical significance level was set at *p*-value <0.05 (two-tailed). Statistical analyses were conducted by R, version 3.6.4 (R Foundation).

## Results

### Descriptive data for characteristics of the participants

Study characteristics of the cohort are presented in Table 1. Of 149,005 individuals, 35,603 [18,567 boys (52.15%)] were exposure to SHS while pregnant, 113,402 [59,452 boys (52.43%)] were NS exposed, with a prevalence of prenatal SHS exposure of 23.89%. The proportion of mothers and fathers with college and above education (>12 years) in the NS exposed group were 60.64 and 61.83%, which were significantly higher than those in the SHS exposed group (54.76, 49.65%). Mean (SD) age of preschoolers was 4.52 (0.78) years for SHS exposed group, 4.47 (0.79) years for the NS exposed group. The LDCDQ scores and rates of suspected DCD by different exposed groups are shown in Supplementary Tables 1, 3. The prevalence of suspected DCD was significantly higher in prenatal SHS-exposed group (16.38%) than in NS-exposed group (14.19%), *P* < 0.001.

### Preschoolers' neurodevelopment outcomes

The sub-category and total LDCDQ scores by gender and age were shown in Table 2. With the increase of age, the mean total scores of LDCDQ of both boys and girls increased gradually, from 66.10 ± 9.43 to 68.48 ± 8.48, and from 67.33 ± 9.01 to 69.70 ± 7.89, respectively. In the same age group, the girls had higher scores in CDM, FM, GC, and total score of LDCDQ than those of boys. According to China norms established previously, the rate of suspected DCD in each group was between 13.70 to 15.96% after gender and age stratification. Boys aged 5 years had the lowest risk of suspected DCD (13.70%), and girls aged 3 years had the highest risk (15.96%) than any other groups.

**Table 2:** The LDCD scores and suspected DCD by gender and age group (*N* = 149,005).

		**Boys**			**Girls**		
		**3**	**4**	**5**	**3**	**4**	**5**
Age (Year)	CDM*	22.62 (3.21)	23.01 (3.04)	23.25 (2.92)	22.67 (3.17)	23.09 (2.93)	23.32 (2.83)
	FM*	21.77 (3.49)	22.59 (3.15)	23.12 (2.92)	22.49 (3.19)	23.25 (2.76)	23.62 (2.56)
	GC*	21.71 (3.49)	22.17 (3.20)	22.39 (3.14)	22.15 (3.19)	22.58 (3.01)	22.76 (2.96)
	Total LDCDQ*	66.10 (9.43)	67.77 (8.85)	68.48 (8.48)	67.33 (9.01)	68.92 (8.22)	69.70 (7.89)
Suspected DCD, *N* (%)	
	Yes	**3,263 (14.29)**	**4,604 (14.49)**	**3,207 (13.70)**	**3,385 (15.96)**	**4,438 (15.39)**	**3,024 (14.43)**
	No	19,571 (85.71)	27,172 (85.51)	20,202 (86.30)	17,820 (84.04)	24,390 (84.61)	17,929 (85.57)

* CDM, Control during movement; FM, Fine motor; GC, General coordination; LDCDQ, Little developmental coordination disorder questionnaire.

### Association of prenatal SHS exposure with sub and total scores of the LDCDQ and potential modifiers

Supplementary Table 1 shown that there was interaction between prenatal SHS exposure and postnatal SHS exposure. Table 3 shown that whether there was postnatal exposure to SHS or not, maternal prenatal SHS exposure had a negative impact on various scores of LDCDQ. However, in all 4 models, the negative impact of prenatal SHS exposure on various scores in the postnatal SHS exposure group was weakened and became statistically non-significant with the CDM score. In the postnatal NSE group, prenatal SHS exposure had the strong negative association with the total score of LDCDQ (β:−0.93, 95% CI:−1.17,−0.69) preschoolers, after adjusting for parental, neonatal, and preschooler characteristics (model 4 in Table 3).

**Table 3:** Association of prenatal SHS exposure with sub and total scores of the LDCDQ, postnatal SHS-exposed stratification analysis (*N* = 149,005, β, 95% CI).

**Models**	**Little DCDQ items**	**All population**	**Postnatal NSE^#^**	**Postnatal SHS^#^**
		**(*N* = 149,005)**	**(*N* = 53,344)**	**(*N* = 61,777)**
Model 1^a^	CDM	−0.23 (-0.26,−0.19)***	−0.22 (-0.31,−0.14)***	−0.04 (-0.09, 0.00)
	FM	−0.29 (-0.33,−0.25)***	−0.28 (-0.36,−0.19)***	−0.07 (-0.12,−0.01) *
	GC	−0.47 (-0.51,−0.43)***	−0.49 (-0.58,−0.40)***	−0.20 (-0.26,−0.15) ***
	Total	−0.99 (-1.09,−0.88)***	−0.99 (-1.24,−0.74)***	−0.31 (-0.46,−0.17) ***
Model 2^b^	CDM	−0.13 (-0.16,−0.09)***	−0.21 (-0.29,−0.12)***	−0.05 (-0.1, 0.00)
	FM	−0.19 (-0.22,−0.15)***	−0.26 (-0.35,−0.18)***	−0.07 (-0.12,−0.02)**
	GC	−0.34 (-0.37,−0.30)***	−0.46 (-0.54,−0.37)***	−0.20 (-0.25,−0.15)***
	Total	−0.65 (-0.75,−0.54)***	−0.93 (-1.17,−0.69)***	−0.32 (-0.46,−0.18)***
Model 3^c^	CDM	−0.12 (-0.16,−0.08)***	−0.20 (-0.29,−0.12)***	−0.04 (-0.09, 0.00)
	FM	−0.18 (-0.22,−0.14)***	−0.25 (-0.34,−0.17)***	−0.07 (-0.12,−0.02)**
	GC	−0.33 (-0.37,−0.29)***	−0.45 (-0.54,−0.36)***	−0.20 (-0.25,−0.15)***
	Total	−0.63 (-0.74,−0.53)***	−0.90 (-1.15,−0.66)***	−0.31 (-0.45,−0.17)***
Model 4^d^	CDM	−0.20 (-0.29,−0.12)***	−0.21 (-0.29,−0.12)***	−0.04 (-0.09, 0.01)
	FM	−0.27 (-0.35,−0.18)***	−0.27 (-0.35,−0.18)***	−0.06 (-0.11,−0.01)*
	GC	−0.46 (-0.54,−0.37)***	−0.45 (-0.54,−0.37)***	−0.19 (-0.24,−0.14)***
	**Total**	-0.93 (-1.17,−0.69)***	-0.93 (-1.17,−0.69)***	-0.30 (-0.43,−0.16)***

# ostnatal NSE, no postnatal second-hand smoke exposure; postnatal SHS, postnatal second-hand smoke exposure.

a Crude model without adjusting.

b Adjusted for maternal age at birth, maternal education, paternal education, parity, maternal comorbidities and pregnancy complications, paternal age at birth, annual per capita household income.

c Further adjusted for gestational age, delivery model, neonatal birth weight, Neonatal asphyxia, and residence history of NICU.

d Further adjusted for sex, currently age, and BMI of preschoolers, medical history of movement disorders, psychiatric medication history and interaction item between SHS and Post SHS.

* *P* < 0.05,

** *P* < 0.01,

*** *P* < 0.001.

CDM, Control during movement; FM, Fine motor; GC, General coordination; LDCDQ, Little developmental coordination disorder questionnaire.

### Associations of prenatal SHS exposure with risk of suspected DCD

The results of the logistic regression crude models in Table 4 shown that maternal prenatal SHS exposure increased the risk of suspected DCD in each model (OR/AOR > 1), but this effect was interfered by postnatal SHS exposure, which with decreased AOR and their 95% CI, spanned 1. Postnatal exposure has a great impact on the risk of suspected DCD.

**Table 4:** Association of prenatal SHS exposure with risk of suspected DCD (*N* = 149,005, ORs/AORs, 95% CI).

**Models**	**All population (*N* = 149,005)**	**Postnatal NSE^#^** **(*N* = 53,344)**	**Postnatal SHS^#^ (*N* = 61,777)**
Prenatal SHS^a^	1.18 (1.15, 1.22)***	1.14 (1.05, 1.23)**	1.03 (0.99, 1.08)
Prenatal SHS^b^	1.10 (1.06, 1.13)***	1.13 (1.04, 1.23)**	1.04 (0.99, 1.09)
Prenatal SHS^c^	1.09 (1.05, 1.13)***	1.13 (1.04, 1.22)**	1.04 (0.99, 1.08)
Prenatal SHS^d^	1.12 (1.03, 1.22)**	1.12 (1.03, 1.22)**	**1.03 (0.99, 1.08)**

# Postnatal NSE: no postnatal second-hand smoke exposure; postnatal SHS: postnatal second-hand smoke exposure.

a Crude model without adjusting for other covariates.

b Adjusted for maternal age at birth, maternal education, paternal education, parity, maternal comorbidities and pregnancy complications, paternal age at birth, annual per capita household income.

c Further adjusted for gestational age, delivery model, neonatal birth weight, neonatal asphyxia, and residence history of NICU.

d Further adjusted for sex, currently age, and BMI of preschoolers, medical history of movement disorders, psychiatric medication history and interaction item between SHS and Post SHS.

** *P* < 0.01,

*** *P* < 0.001.

### Sensitivity analyses

After applying the constraints, we generated a data containing 58,630 participants for sensitivity analysis. The pattern of results for the associations between prenatal SHS exposure and the preschoolers' motor competence remained robust when adjusting, or not adjusting for, covariates (Supplementary Figures 1, 2).

## Discussion

Based on a national representative population sample of preschoolers in China, this study provides evidence of an elevated risk of suspected DCD associated with maternal prenatal SHS exposure. Girls had higher scores in all three sub scores, and the total score of LDCDQ than boys in the same age group. The risk of suspected DCD tends to decrease with age in both boys and girls in the general population. Furthermore, our analysis suggests that parental, neonatal, and preschooler factors had comparable or even larger estimates of the effect than SHS for suspected DCD, especially the postnatal SHS exposure. In the postnatal SHS exposer group, the effect of prenatal SHS exposure was significantly reduced. To our knowledge, this is the first nationwide study that linked prenatal SHS exposure to preschooler risk of DCD. Our findings have important implications in formulating prevention and intervention measures to reduce DCD risk associated with prenatal SHS.

### Epidemiological studies

Previous research has found that prenatal exposure of the human fetus to SHS has been epidemiologically linked to preterm birth, reduced birth weight, enhanced susceptibility to respiratory diseases, and changes in immune response (10, 12, 33, 34). Moreover, studies have also shown a relationship between SHS exposure during pregnancy and neurodevelopment in children. Although the study reported here is based on parent reports, studies that have measured cotinine (a nicotine metabolite) levels in pregnant mothers exposed to SHS, but excluding mothers who actively smoke, demonstrate results consistent with the findings reported here. Researchers found that the cognitive development of infants at 24 months of age decreased significantly with increasing maternal cotinine—in a study examining 720 mother-infant pairs (35). Moreover, cotinine levels during pregnancy have been negatively associated with caregiver reports of their children at 8 years of age (*N* = 239) in their ability to initiate activities, working memory, and ability to organize personal spaces (36).

The findings reported in this large-scale population retrospective cohort study are consistent with previous research with smaller sample sizes. A single center study (*N* = 8,586) in Shanghai, China, found that the occurrence of DCD among children was positively associated with prenatal SHS exposure among mothers (OR = 1.77; 95% CI: 1.47–2.14) (24). Furthermore, a cross-sectional examination of 122 children in Niagara, Ontario, Canada, suggested that exposure to SHS during pregnancy increases the risk of DCD in children (23).

### Biological causation

The toxic effects of SHS may largely depend on its chemical ingredients. Although DCD may result from atypical brain structure and function, the exact cause of DCD is not fully known. However, the cerebellum is a core brain region responsible for motor control and coordination and is considered involved in the motor symptoms of DCD (37). Research by Fuller and colleagues found that the critical postnatal period of cerebellar development is vulnerable to environmental tobacco smoke (ETS) exposure, and the possible mechanism is that developmental ETS exposure disrupts mitochondria and related aerobic pathways (38). Nicotine, as one of the main components in tobacco, has also been found to disrupt the growth of the types of neural cells that are abundant in the cerebellum (39).

Furthermore, an *in-vitro* experiment on the effect of nicotine on locomotor networks showed that nicotine exposure could reduce the activation of signaling pathways and produce toxic effects on central and ventral spinal cord neurons, which corroborates the risk of cigarette smoking in fetal and neonate development (40). The study by Delcour et al. showed with rats that, even in the absence of a brain injury, early locomotor movement restriction can lead to later maladaptive plasticity in the sensorimotor cortex. The resultant movement disorder—consistent with characteristics of DCD—indicates a possible mechanism underlying neurodevelopmental motor impairment (41).

### Sex- and age-dependent difference*s* of suspected DCD

A number of studies reported the possible differences between different sex in movement disorders (42). Age is another variable of interest to DCD researchers. We used the age- and sex-specific norms of the LDCDQ, and cutoff scores were provided based on a national sample in China to indicate suspected impairments of motor coordination (Table 2). And we found increased motor performance and decreased risk of suspected DCD with the increase of age in different gender groups. The risk of suspected DCD decreased with age in girls, which is consistent with the findings of earlier studies (24).

But *age hypothesis* was presented that the activity deficit in children with DCD would grow larger as children's play became more complex and rule-bound (43). In this study, the incidence of DCD in boys reached the highest level at the age of 4, rather than a consistent decrease. Therefore, more attention should be paid to boys of this age and those younger than this age. In addition, our results show that in the same age group, the incidence of suspected DCD in girls was higher than that in boys. Cairney, et al. also found that effect of DCD appears to be particularly serious among females but may diminish with time among males (44).

### Covariates relating to parental, neonatal, and child factors

Previous research results from our research team show that maternal age, parents' education level, single-child status, children's BMI, gender, prematurity, placenta previa, threatened abortion, placental abruption, fetal distress during labor, chronic lung disease, and newborn pathological jaundice are all risk factors for DCD (32, 45). Moreover, a recent scoping review showed that preterm birth and male sex were consistently associated with an increased risk of DCD (21). In the study reported here, we found that in addition to the above factors, we observed the risk of postnatal SHS exposure to the occurrence of suspected DCD in preschoolers. It was obvious from Table 4 that without postnatal SHS exposure, prenatal SHS exposure increased the risk of suspected DCD. However, for children with postnatal SHS exposure, the harmful effects of prenatal exposure were concealed, indicating the postnatal SHS exposure has a greater impact on the risk of suspected DCD. Most population studies have failed to assess the true independent effects of postnatal SHS exposure (46). Animal experiments by Gospe, et al. confirmed that environmental tobacco smoke exposure during pregnancy has less effect on the indices of brain cell number and size in rats than postnatal exposure (47). Even so the risk of prenatal SHS exposure cannot be ignored.

### Strengths and limitations

The large sample size is one of the advantages of this study, which provided sufficient statistical power to study an often under-reported developmental disorder (48). One of the limitations of this study is that we estimated SHS exposure through parental self-report, which can be a less objective and inaccurate measure compared with biomarkers. However, our findings are in line with studies using biomarkers (38, 49) and a meta-analysis of studies that validated self-reported smoking behavior with biochemical measurements concluded that self-reports of smoking status are generally accurate (50). Another limitation is the reliance on the LDCDQ—a parent report tool is not the criterion standard for assessing risk of DCD. Further work should consider replication with measures such as the Movement Assessment Battery for Children, Second Edition (MABC-2), which is a validated, standardized and norm referenced test used to measure motor proficiency in children with DCD (51). In addition, this is a retrospective study, which may have some recall bias. The study only included the public kindergartens, which may also have some selection bias. Although we control the covariates, it is not as persuasive as prospective cohort studies. The multiple situations of prenatal maternal active smoking exposure, SHS exposure, postnatal maternal and paternal smoking exposure, postnatal other family members smoking exposure, and the interaction between these situations need to be discussed in detail in the future design.

## Conclusions

In summary, our findings—which were derived from a large-scale representative population study—provide evidence that maternal prenatal SHS exposure elevates the risk of DCD in preschoolers in China but this effect was interfered by postnatal SHS exposure. The findings align with an emerging view of the neurotoxic effects of tobacco smoke during gestation and point toward health messaging to promote greater awareness of the effects of SHS during pregnancy as well as the role of screening for SHS exposure as an early indicator of motor impairment risk.

## Data availability statement

The raw data supporting the conclusions of this article will be made available by the authors, without undue reservation.

## Ethics statement

The studies involving human participants were reviewed and approved by Ethics Committee of Shanghai First Maternity and Infant Hospital, School of Medicine, Tongji University. Written informed consent to participate in this study was provided by the participants' legal guardian/next of kin.

## Author contributions

MW: conceptualization, methodology, and writing—original draft. GW: writing—review and editing. GC and LZ: project administration, methodology, and data collection. CH: data curation, data cleaning, and analysis. XD: project administration and questionnaire revision. WD: conceptualization, methodology, writing—review and editing, and supervision. JH: supervision, funding acquisition, validation, and writing—review and editing. All authors contributed to the article and approved the submitted version.

## Funding

This study was supported by Shanghai Pudong Municipal Health Commission (PW2020D-11), Shanghai Municipal Health Commission (2020YJZX0213), and Clinical Research Plan of Shanghai Hospital Development Center (SHDC2020CR1047B-003). The funders had no role in the conduct of the study, the analysis or interpretation of data, and the preparation, review, or approval of the manuscript.

## Conflict of interest

The authors declare that the research was conducted in the absence of any commercial or financial relationships that could be construed as a potential conflict of interest.

## Publisher's note

All claims expressed in this article are solely those of the authors and do not necessarily represent those of their affiliated organizations, or those of the publisher, the editors and the reviewers. Any product that may be evaluated in this article, or claim that may be made by its manufacturer, is not guaranteed or endorsed by the publisher.
