# Supplementary material for: Prenatal second-hand smoke exposure and the risk of suspected developmental coordination disorder in preschoolers: A nationwide retrospective cohort study in China
Source: Front Public Health. 2022 Nov 8;10:993471. doi: 10.3389/fpubh.2022.993471 (PMC9686837; doi:10.3389/fpubh.2022.993471)
Supplement: Supplementary file 1 [file Data_Sheet_1.docx]

Supplementary Material

# Supplementary Tables

**Supplemental Table 1** Collinearity Diagnostics of Multivariate Regression Model

| Variable | Df* | GVIF* |
| --- | --- | --- |
| SHS exposure | 1 | 1.01 |
| Maternal education | 4 | 1.02 |
| Maternal comorbidities and pregnancy complications | 1 | 1.01 |
| Maternal age at birth | 3 | 1.14 |
| Paternal age at birth | 3 | 1.14 |
| Household incomes | 9 | 1.01 |
| Parity | 2 | 1.01 |
| Gestational age | 2 | 1.02 |
| Cesarean delivery | 1 | 1.02 |
| Birth weight | 2 | 1.02 |
| Neonatal asphyxia | 1 | 1.01 |
| NICU | 1 | 1.03 |
| BMI | 1 | 1.00 |
| Medical history of movement disorders | 1 | 1.01 |
| Psychiatric medication | 1 | 1.01 |
| Sex | 1 | 1.00 |
| Current Age | 1 | 1.00 |
| Postnatal SHS exposure | 1 | 1.09 |

*Df: Degrees of freedom, GVIF: generalized variance-inflation factors.

**Supplemental Table 2** Effects of Postnatal SHS Exposure on Little DCDQ Items and Suspected DCD

| Variable | Item | CDM^#^ | FM^#^ | GC^#^ | Total^#^ | DCD^##^ |
| --- | --- | --- | --- | --- | --- | --- |
| Post SHS | Estimate (95%CI) | -0.05 (-0.09, -0.01) | -0.12 (-0.16, -0.08) | -0.14 (-0.19, -0.1) | -0.31(-0.43, -0.2) | 1.06 (1.02, 1.10) |
|  | *P* | 0.017 | <0.001 | <0.001 | <0.001 | 0.005 |
| SHS **·** Post SHS * | Estimate (95%CI) | 0.17 (0.07, 0.26) | 0.21 (0.11,0.3) | 0.26(0.16, 0.36) | 0.63 (0.36, 0.91) | 0.92 (0.84, 1.01) |
|  | *P* | 0.001 | <0.001 | <0.001 | <0.001 | 0.074 |

*: Interaction item between SHS and Post SHS.

#: Coefficients (95%CI) of multiple linear regression.

##: Odds Ratio (95%CI) of logistic regression.

**Supplemental Table 3** The LDCDQ Scores and rates of suspected DCD by different prenatal exposed groups

| **Variable** | | **M (SD) / N (%)** | | ***P*** |
| --- | --- | --- | --- | --- |
|  |  | **SHS-exposed group ^*^(n=****35603)** | **NS-exposed group^*^ (n=113402)** |  |
| CDM^*^, M (SD) | | 22.83 (3.09) | 23.05 (3.01) | <0.001 |
| FM^*^, M (SD) | | 22.59 (3.17) | 22.88 (3.05) | <0.001 |
| GC^*^, M (SD) | | 21.93 (3.24) | 22.41 (3.13) | <0.001 |
| Total LDCDQ^*^, M (SD) | | 67.35 (8.90) | 68.34 (8.67) | <0.001 |
| Suspected DCD, N (%) | |  |  |  |
|  | Yes | 5831 (16.38) | 16090 (14.19) | <0.001 |
|  | No | 29772 (83.62) | 97312 (85.81) |  |

* CDM: Control during movement, FM: Fine motor, GC: General coordination, LDCDQ: Little developmental coordination disorder questionnaire

# Supplementary Figures


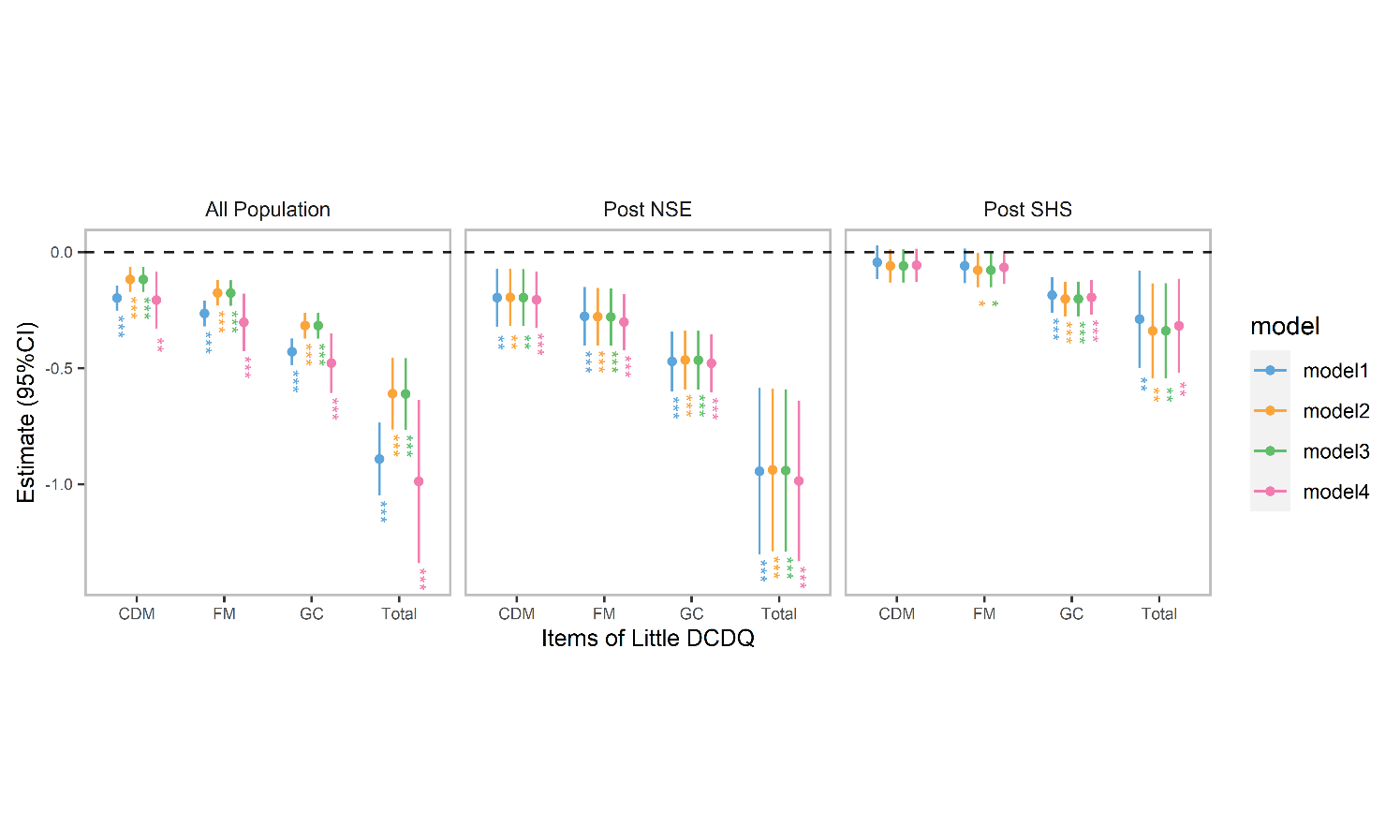


**Supplementary Figure 1.** Association of SHS exposure with subscores of the LDCDQ, in a restricted population (N=58630).


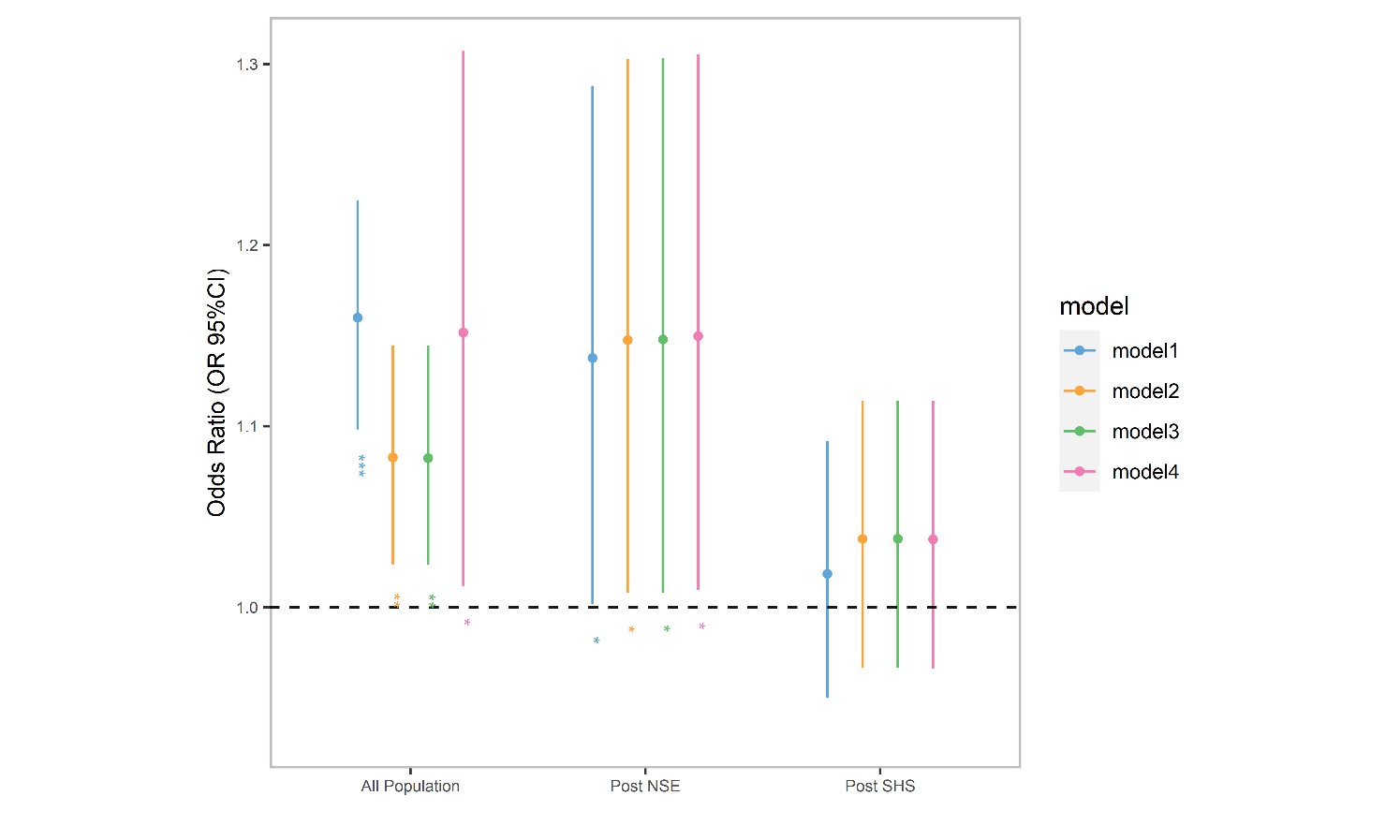


**Supplementary Figure 2.** Association of SHS exposure with risk of suspected DCD, in a restricted population (N=58630).
